# Zebrafish Larvae as a Behavioral Model in Neuropharmacology

**DOI:** 10.3390/biomedicines7010023

**Published:** 2019-03-26

**Authors:** Ram Manohar Basnet, Daniela Zizioli, Somrat Taweedet, Dario Finazzi, Maurizio Memo

**Affiliations:** 1 Department of Molecular and Translational Medicine, University of Brescia, 25123 Brescia, Italy; daniela.zizioli@unibs.it (D.Z.); s.taweedet@unibs.it (S.T.); dario.finazzi@unibs.it (D.F.); maurizio.memo@unibs.it (M.M.); 2 Clinical Chemistry Laboratory, ASST-Spedali Civili di Brescia, 25123 Brescia, Italy

**Keywords:** zebrafish larvae, behavior, neuropharmacology, high-throughput screening, neuroactive drugs, light-dark test

## Abstract

Zebrafish larvae show a clear and distinct pattern of swimming in response to light and dark conditions, following the development of a swim bladder at 4 days post fertilization. This swimming behavior is increasingly employed in the screening of neuroactive drugs. The recent emergence of high-throughput techniques for the automatic tracking of zebrafish larvae has further allowed an objective and efficient way of finding subtle behavioral changes that could go unnoticed during manual observations. This review highlights the use of zebrafish larvae as a high-throughput behavioral model for the screening of neuroactive compounds. We describe, in brief, the behavior repertoire of zebrafish larvae. Then, we focus on the utilization of light-dark locomotion test in identifying and screening of neuroactive compounds.

## 1. Introduction

There are many practical advantages of using zebrafish as an animal model such as external fertilization, optical transparency, small size, high fecundity, and low housing cost [1,2,3]. The zebrafish genome has been completely sequenced and the majority of zebrafish genes are common to humans with 84% of genes known to be associated with human disease having zebrafish counterparts [4]. Zebrafish also have a high homology to mammalian morphology and biology making them an attractive animal model in studying human disorders [5]. The validity of zebrafish models is not only due to the closeness of zebrafish morphologically and genetically, but also behaviorally. In particular, zebrafish exhibit a wide range of complex behaviors including social, anxiety, learning and memory, and defensiveness that may be useful for modeling neurological and psychiatric diseases [6,7,8,9,10].

The mammalian and zebrafish behavioral paradigms are in close parallel suggesting the evolutionarily conserved nature of many behaviors across species [3,5,11]. Despite neuroanatomical differences between the mammals and teleosts, evidence shows homologous functions in several key zebrafish brain areas [12,13,14,15]. The recent emergence of high-throughput tracking of zebrafish embryos and larvae has further bolstered the behavior research in the zebrafish field, making the research more objective, replicable, and efficient. Increased research in zebrafish has led to the characterization of a wide range of behaviors at different developmental stages. A comprehensive catalog of zebrafish describing more than one hundred behaviors of embryonic larvae and adult zebrafish is already available [16]. 

In this review, we will concentrate on the behavior of zebrafish larvae. Zebrafish larvae show similarity to adult zebrafish and other vertebrates in key behaviors utilized in neuropharmacology such as anxiety and stress [17]. The small size, optical transparency, rapid generation time, easy manipulation, permeability to small molecules, and cost effectiveness are some general advantages of larval zebrafish over the adult zebrafish. Lately, with the advent in technology, the use of zebrafish larvae is gaining momentum in the field of behavioral research, particularly in neuropharmacology and circuit neuroscience [18,19]. For instance, a large-scale high-throughput behavioral analysis, which could screen thousands of compounds in a day, is possible using zebrafish larvae [19,20]. Similarly, it is also possible to visualize/monitor/track the neuronal activity of zebrafish larvae while the larvae are engaged in a behavior [16,19,20,21]. Besides the technical aspects, the use of zebrafish larvae follows the principles of 3Rs (Replacement, Reduction, Refinement) [21], in which there is an effort to reduce the use of adult animals in biomedical research [22]. However, the use of zebrafish larvae in behavior research suffers from some limitations. For instance, the behavior repertoire of zebrafish larvae is not as sophisticated as that of adult zebrafish and other vertebrates [23]. Various factors such as the size and color of individual wells affect the behavior in zebrafish larvae [23]. Overall, the use of larvae has some limitations but the advantages as described above make them an attractive model in the field of biomedical research. 

It is well known that zebrafish larvae are sensitive to a variety of stimulus modalities, including touch, olfaction, chemosensation, audition, vestibular inputs, heat, and vision [24]. Among the behaviors, locomotion or swimming in zebrafish larvae is increasingly gaining attention in neuropharmacology. Swimming starts in zebrafish at 48–72 h post fertilization (hpf) following hatching although zebrafish embryos as early as 27 hpf, when dechorionated, can swim in response to touch [25]. After hatching, the newly hatched larvae show burst swimming, which then matures to beat and glide swimming by 4 days post fertilization (dpf) [26,27,28]. Zebrafish larvae after 4 dpf, when exposed to an alternating light and dark stimuli, create a pattern of increased movement in the dark followed by resting state in the light [29]. This behavior has been increasingly utilized in the high-throughput screening of various neuroactive drugs recently. This review focuses on the light and dark stimuli-induced locomotor behavior of zebrafish larvae as a behavioral model to study neuroactive compounds. We describe, in brief, the behavior repertoire of zebrafish larvae and then discuss the utility and importance of light and dark stimuli-induced locomotor behavior in identifying neuroactive compounds. In addition, we also discuss the recent progress made in behavioral research in the context of the emergence of various high-throughput techniques for the automatic tracking of larvae.

## 2. Connection Between Brain and Behavior

Zebrafish provide a platform among vertebrate model systems to study the connection between different brain areas with behavior. The structural organization and cellular morphology of the zebrafish brain is very similar to that of other vertebrates, including chickens, rats, and humans [5,30,31]. Many structures, such as retina, olfactory bulb, cerebellum, and spinal cord, are similar in architecture in zebrafish and other vertebrates [18]. Besides the structural homology, the zebrafish brain is similar to humans and rodents with respect to neurochemistry [5]. The zebrafish brain contains all the major components required for neurotransmission, such as neurotransmitter receptors, transporters, and enzymes of synthesis and metabolism. It also has the same neurotransmitter system as that of higher vertebrates such as GABA, glutamate, dopamine, noradrenaline, serotonin, histamine, and acetylcholine [5,32]. Among the various neurochemical pathways, several pathways involved in the modulation of behavior in zebrafish have been well characterized. For example, the ascending midbrain dopaminergic pathway has been well characterized [33] with a number of putative functional homologues with their connection and projections. Dopaminergic neurons are detected at 18-19 hpf in the ventral diencephalon which ascend in the striatum and resemble a mammalian nigrostriatal system [32].

Also, many brain regions associated with human disease show molecular and structural homology in zebrafish [34]. For example, the habenula and amygdala in zebrafish are involved in the control of affective behaviors, as in humans and rodents [5]. The habenula regulates the release of serotonin and dopamine and is evolutionarily highly conserved [10]. The hyperactivation of habenula leads to similar conditions such as depression and stress-related behaviors in both mammals and zebrafish [5]. Habenular hyperactivation causes depression in humans and rodents and induces stress-related behaviors in zebrafish. This demonstrates the similarity in brain function between zebrafish and mammals [5]. However, one difference with mammals is the lack of neocortex in zebrafish—the primary area controlling executive functions commonly disrupted in psychiatric disease. Recently, it has been shown that the smaller vertebrate brain such as in zebrafish, despite lacking neocortex, is capable of cognitive processing and even complex decision making [35]. 

In addition to structural homology, zebrafish larvae have an added advantage of visualizing the neural circuits while they perform a certain behavior. The larval brain at 5 dpf is very compact at 1.5 mm in length and 500 μm in thickness, thus making virtually all the neurons accessible to in vivo imaging [15,18,36,37]. The visualization of brain activity through imaging methods is an important step to understand how the brain contributes to normal and abnormal behavior [18]. The emergence of circuit neuroscience has provided opportunity to study the neural circuits involved in various behaviors, such as prey capture, optokinetic response, and optomotor response in zebrafish larvae [18]. 

## 3. Behavior Repertoire in Zebrafish Larvae

The increased characterization of behaviors in zebrafish larvae, which are comparable to the adult zebrafish and rodents, have increased the scope of their utility in the field of neuroscience [5,16]. The larval stage of zebrafish starts from 72 hpf (from protruding mouth stage) and lasts until they undergo metamorphosis to become juvenile [38] at approximately one month. During this period, zebrafish possess a plethora of behaviors which could be analyzed automatically or manually and quantitatively or qualitatively. The zebrafish behaviors have been exploited in diverse research areas such as ethology, toxicology, pharmacology, neuroscience, and genetics [39,40,41,42,43,44,45]. In neuropharmacology, the behavior repertoire of zebrafish larvae has been utilized in a multipurpose way—for identifying new drugs with central nervous system (CNS) effect, for repurposing of drugs with CNS effects, for identifying drugs for various psychiatric illnesses such as anxiety and mood disorders, for the study of drugs affecting sleep disorders, and for identifying the therapeutic and target specificity of newer compounds [19,46,47,48]. The commonly exploited behaviors of zebrafish larvae are described below. 

### 3.1. Thigmotaxis

The tendency of an animal to move in contact with a vertical surface is called thigmotaxis [49]. In thigmotaxis, an animal avoids the center of an arena and moves towards the edge or periphery of a novel environment such as a wall [50]. Therefore, it is also known as wall-hugging behavior. It is a valid index of anxiety [50,51] and is evolutionarily conserved across different species, such as fish, rodents, and humans [52,53]. Zebrafish larvae with thigmotaxis prefer to stay near the wall of multi-well plates or Petri dishes. They show thigmotaxis as early as 5 dpf [53]. Anxiolytic drugs, such as diazepam, and anxiogenic drugs, such as caffeine and pentylenetetrazole, have been shown to attenuate and enhance thigmotaxis in larval zebrafish [53,54,55]. Therefore, thigmotaxis is crucial in studying anxiogenic and anxiolytic drugs. 

### 3.2. Startle Response

The startle response is a sensorimotor response universal in animals in which they show a rapid and protective response to sudden, strong, and abrupt stimulus such as loud sounds and unexpected touch [56]. Zebrafish larvae show a sudden increase in the velocity and acceleration of movement/swimming in response to visual, tactile, or acoustic stimuli [51]. The startle response is important as it provides the status of the integration of sensory and motor stimulus. The tactile startle response is present as early as 2 dpf and it can be induced by touching the head or tail of the zebrafish larvae [51]. The visual startle response develops by 3 dpf. In the visual startle response, larval zebrafish are placed inside a closed chamber equipped with an infrared light and camera. After a brief period of adaptation, they are presented with transient white light and the resulting movement is documented to measure the visual startle response. The test to measure the visual startle response is also called the visual motor response test [57]. Zebrafish larvae show the acoustic startle response after 5 dpf [58]. They show a startle response to sound frequencies of greater than 200 Hz [59]. The startle response is vital in the study of neuropsychiatric diseases of humans as an abnormal startle response signals a broader neurological problem [56]. Study of the startle response plays a major part in neuropharmacological research as some drugs that alter prepulse inhibition could be used for the treatment of diseases such as schizophrenia [60]. 

### 3.3. Optokinetic Response

The optokinetic response is the stereotyped eye movement in response to movement in the field of vision. It helps in the stabilization of images in the retina. It is also important in maintaining visual acuity, which in turn helps animals in spatial orientation, hunting for prey, and escaping from predators [61]. Zebrafish larvae develop optokinetic response by 4 dpf [62], following which it can be precisely measured [63]. The optokinetic response is evoked in zebrafish larvae by movements in the form of moving graphics on a liquid crystal display (LCD) monitor [17] or a striped black and white rotating drum [61]. Many drugs acting on the central nervous system, such as sedatives, anti-psychotics, and anti-depressants, modulate eye movements. Therefore, the optokinetic response can be used as a complementary test for characterizing the central nervous system effects of drugs [64]. 

### 3.4. Optomotor Response

When zebrafish larvae are presented with moving stripes, they tend to swim in the same direction as the moving stripes. This visual-motor behavior of zebrafish is called the optomotor response. Zebrafish larvae start displaying this behavior at 6 dpf [65,66]. This response can be used to test visual function of the zebrafish larvae and testing of a compound at an early stage of discovery using a low volume of the compound [67].

### 3.5. Habituation

Habituation is a non-associative form of learning in which a repeated stimulus leads to an attenuated response [58,68]. It is evolutionarily conserved and present in a wide range of species from invertebrates, such as Aplysia and Drosophila, to vertebrates such as rodents [58]. It serves as a mechanism by which the nervous system filters irrelevant stimuli. Zebrafish larvae at 6 dpf have been demonstrated to show habituation. Defective habituation is associated with several neuropsychiatric diseases such as schizophrenia and attention deficit hyperactivity dis//Rihel, 2010 #75\\order (ADHD) [69]. Habituation studies could be used for the high-throughput screening of new compounds with specific effects on non-associative learning [69]. 

### 3.6. Prey Capture

Once the zebrafish embryos are hatched, they initiate swimming and catch any potential food, also called prey capture. This behavior develops in zebrafish larvae as early as 4 dpf [70]. Prey capture is a complex locomotor behavior and the larvae use vision along with fine axial motor control to find and follow the prey [71,72]. It can be elicited in zebrafish larvae by placing a small air bubble in the test arena. The reaction of zebrafish larvae to the air bubble involves recognition, approach, decision making, and capturing [70]. Prey capture is crucial for the survival of the zebrafish larvae post absorption of yolk sac. As prey capture involves a decision making process, which is directly related to cognition [70], it could be used for the screening of drugs affecting cognition. 

### 3.7. Sleep/Awake Behavior

The sleep/awake pattern of zebrafish is similar to that of humans [73,74]. Zebrafish larvae from 6 to 10 dpf have been used for sleep research. Similar to human infants, larval zebrafish at 6–10 dpf showed a higher percentage of sleep compared to the adult zebrafish [73]. The sleep/awake pattern of zebrafish larvae has been exploited for studying drugs affecting the nervous system [75,76,77,78]. Sigurgeirsson et al. characterized the effects of modafinil—a narcoleptic drug—on sleep–wake cycles in larval zebrafish. Using 24 h behavioral monitoring, the effects of modafinil—a drug used for the treatment of narcolepsy—on sleep/awake length and structure of bout distributions were evaluated both in day and night. Modafinil had similar effects in larval zebrafish as it does in mammals in the sleep/awake cycles [77]. Wang et al. showed that tracking of the normal movement behavior of zebrafish larvae using a video-tracking system can be utilized for determining the effect of anti-depressant drugs [78]. Similarly, Rihel et al. also performed a detailed analysis of multiple behavioral parameters using an automated rest/awake behavioral assay in zebrafish larvae for the screening of 5648 compounds. This assay was able to identify 547 compounds which significantly altered the larval behavior as compared to controls [75]. Therefore, the zebrafish rest/awake cycle could potentially be utilized in identifying or prescreening of the neuroactive drugs. 

### 3.8. Locomotor Behavior

**Figure 1:**
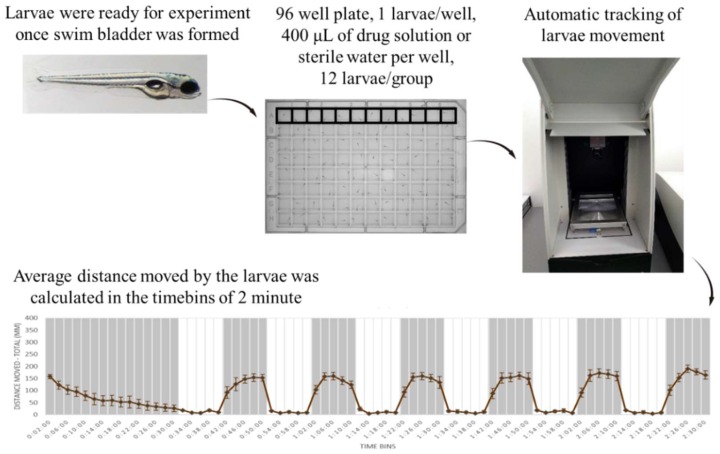
General scheme of the light-dark locomotion test. Zebrafish larvae were placed in a 96-well plate with one larva per well and 300 µL of fish water. The plate containing the larvae was then placed in a Danio vision observation chamber following which the high-throughput tracking of zebrafish larvae was performed. The experiment duration was 2 h and 30 min and consisted of: 30 min of acclimatization, followed by 6 cycles of alternating light and dark periods. Each point in the graph represents the mean ± SEM (standard error of mean) of the distance moved by zebrafish larvae in 2 min of time bins. The shaded part represents the dark period and the unshaded part represents the light period. The total number of embryos used was 12 (*n* = 12).

Locomotion is integral for the survival of animals. Zebrafish larvae show mature swimming at 4–5 dpf [79] following the development of a swim bladder. Locomotion in zebrafish is a complex behavior produced by the activity of various neurons—reticulospinal neurons of the brain stem along with descending vestibulospinal or neuromodulatory projections [80]. All these pathways are evolutionarily conserved among the vertebrates [80]. In addition, the neurotransmitter systems present in the zebrafish are also common to that of other vertebrates [81]. The precision analysis of locomotion in zebrafish larvae with high-throughput methods could provide accurate information needed for the screening and identification of neuroactive compounds. There are various locomotor behavioral assays used for identifying neuroactive drugs [17]. Among them is the light-dark locomotion test in which the locomotor activity and movement pattern of zebrafish larvae are analyzed via high-throughput automatic tracking by placing them in multi-well plates inside a closed chamber and exposing them to alternating light and dark conditions following a period of acclimatization, as shown in Figure 1. The distance travelled and the pattern of movement of zebrafish larvae in each of the conditions is evaluated for understanding the neurobehavioral effects. Zebrafish larvae show a specific pattern of movement when exposed to alternating light and dark conditions [82]. The movement pattern depends on the transitions—light to dark transition and dark to light transition. The light-dark transition increases locomotor activity whereas the dark-light transition decreases locomotor activity in zebrafish larvae. The increased levels of locomotor activities upon the light-dark transition are attributed to the increased stress/anxiety level in zebrafish larvae [83,84,85]. These locomotor activities depend on the integrity of brain function, nervous system development, and visual pathways [86,87]. 

## 4. Identification of Neuroactive Compounds

### 4.1. Light-Dark Locomotion Test

The locomotor activity and pattern of zebrafish larvae in light and dark conditions have been used for high-throughput testing and screening of various substances for their neuroactive properties [83]. There are variations in the protocols used for performing the light-dark locomotion test [83,88,89]. The variations are in the stage of zebrafish larvae, number of wells in multi-well plates, and the length of experiments including the length of light and dark conditions, as shown in Table 1.

**Figure 2:**
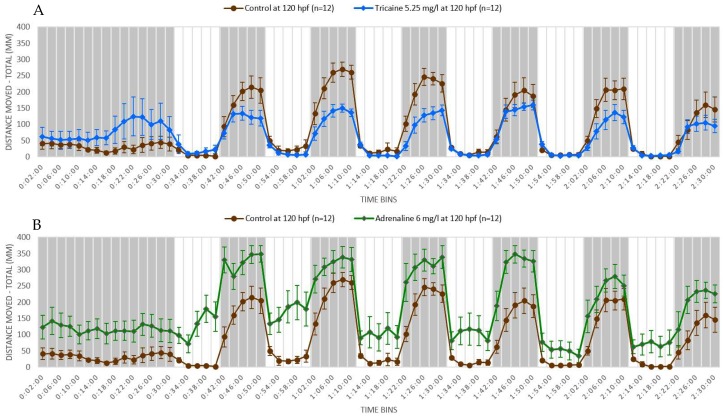
Swimming pattern of AB wild type zebrafish larvae after drug treatment. Zebrafish larvae (*n* = 12 for each drug) at 5 dpf were exposed to sterile water (used as control), adrenaline 0.03 mM (6 mg/L), and tricaine 0.02 mM (5.25 mg/L). The total volume of drug solution or sterile water used was 300 µL per well. The exposure was started half an hour before the experiment and continued till the conclusion of the experiment. The experiment protocol was the same as described in Figure 1. The results were calculated as mean ± SEM of the distance moved by each experimental group in 2 min of time bins. Adrenaline-treated zebrafish larvae showed increased movement throughout the experiment (**A**). On the contrary, tricaine-treated zebrafish larvae showed decreased movement throughout the experiment (**B**). *n* = 12 for each experimental group.

We performed a light-dark locomotion test in which zebrafish larvae at 5 dpf were exposed to two known neuroactive compounds—adrenaline and tricaine. Adrenaline is a non-selective agonist of adrenergic receptors acting as a main neurotransmitter in the autonomic nervous system [105]. Tricaine is a local anesthetic that is specifically used to induce anesthesia and euthanasia in fish. Tricaine induces nervous system suppression by blocking the sodium channels and thereby inhibiting the membrane excitability [106]. We found that adrenaline-treated larvae showed increased locomotor activity throughout the light and dark periods. Also, the response in the adrenaline-treated larvae was more robust than that of controls following the light-dark or dark-light transitions, as shown in Figure 2A. The effect of tricaine was opposite to that of adrenaline in that tricaine-treated larvae showed decreased locomotor activity in the dark condition, although the locomotor activity was similar to control in the light condition, as shown in Figure 2B. Also, the responses to dark-light and light-dark transition were attenuated in the tricaine-treated larvae. Adrenaline is a neuro-stimulant and its exposure increased the locomotor activity and aggravated the startle response in zebrafish larvae, whereas tricaine—a CNS depressant—decreased the locomotor activity and attenuated the startle response in zebrafish larvae. These stimulatory and depressant responses elicited by neuroactive drugs in zebrafish larvae can be exploited in high-throughput screening to identify neuroactive compounds. Thus, zebrafish larvae are sensitive to light and dark conditions and transition from light to dark conditions and vice versa. Treatment of zebrafish larvae with neuroactive substances could change the response pattern of the larvae, which could then be used to determine whether a substance has a neuroactive property or not. 

### 4.2. Neuroactive Drugs

Using d-amphetamine, cocaine, and ethanol, Irons et al. showed that zebrafish larvae are sensitive to neuroactive drugs and their locomotor response is like that of mammals [83]. Cocaine and low-dose amphetamine increased locomotor activity in zebrafish larvae, which is similar to their effects in humans where they act as a CNS stimulant and increase motor activity. Zebrafish larvae treated with low-dose ethanol (1%) showed an increased locomotor activity [83,84,96,97] whereas those treated with higher dose ethanol showed a decreased locomotor activity as compared with the controls. This effect of ethanol in zebrafish larvae is similar to that in humans, where low-dose ethanol acts as a CNS stimulant, whereas high-dose ethanol act as a CNS depressant [107].

Convulsant drugs, such as pentylenetetrazole and picrotoxin, had similar effects. They caused dose-dependent increases in locomotor activity in zebrafish larvae. At lower concentrations, they caused increased locomotor activity in the dark condition, whereas at the higher concentrations they induced a reversal of normal patterns of activity in zebrafish larvae, i.e., a dark-light transition eliciting an increased activity and a light-dark transition eliciting a decreased activity [54,90,100]. The similar responses of PTZ and picrotoxin in zebrafish larvae could be due to their similar mechanisms of action as both of them are GABA receptor antagonists. Similarly, convulsant drugs such as 4-aminopyridine, a potassium channel blocker and aconitine, a voltage gated sodium channel agonist, also induced increased locomotor activity in zebrafish larvae in the dark condition [90]. Thus, the convulsant drugs showed similar effects in zebrafish larvae. In particular, they increased locomotor activity, specifically in the dark condition of the light-dark locomotion test. Besides, most of these drugs also induced a robust startle response following the light to dark transition and the larvae took longer time to return to the baseline movement following the dark to light transition [54,90,100]. 

The effect of anticonvulsant drugs in the zebrafish larvae was expectedly opposite to that of convulsant drugs. Antiepileptic drugs such as diphenylhydantoin (DPH) and sodium valproic acid showed a decrease in locomotor activity in zebrafish larvae in both the dark and light conditions [52]. DPH is a sodium channel blocker and acts by modifying the glutamatergic transmission [108]. Studies done in rodents have shown that DPH blocks the increase of locomotor activity induced by methylphenidate treatment or electroconvulsive shock [109,110]. Sodium valproate acts by increasing the level of GABA, inhibiting voltage-gated sodium channels [8], and modulating the NMDA receptor-mediated actions [111]. MK 801 (dizocilpine)—an NMDA receptor antagonist—decreased the movement in zebrafish larvae in both the light and dark conditions. The NMDA receptor is required for the glutamate-induced excitatory neurotransmission. The decrease in movement in larvae treated with MK 801 could be due to the inhibition of excitatory neurotransmitter glutamate [99]. MK 801 also acts as a potent anticonvulsant and similar to DPH and sodium valproate, it decreased the locomotor activity in zebrafish larvae [99]. Therefore, the neurodepressant effect of anticonvulsant drugs is confirmed by the marked decrease in locomotor activity in zebrafish larvae.

Yohimbine—an alpha 2 adrenergic receptor antagonist—increased the locomotor activity in the dark condition [99], but at higher concentrations, it reduced the locomotor activity in both the light and dark conditions [82,99]. This effect of yohimbine in zebrafish is similar to that observed in rodents. The decrease in locomotor activity observed at higher concentrations could be due to the alpha1 adrenergic receptor antagonism [82]. Similarly, venlafaxine—a serotonin-norepinephrine reuptake inhibitor—also induced a decrease in locomotor activity in the dark condition in zebrafish larvae [104]. However, this effect of venlafaxine in zebrafish larvae is opposite to that observed in rodents, where it was shown to increase the locomotor activity [112,113].

Drugs affecting dopaminergic receptors showed an altered locomotor activity in a dose-dependent manner in the light-dark locomotion test in zebrafish larvae. Selective dopamine agonists like SKF-38393 and quinpirole increased the locomotor activity and selective dopamine antagonists like SCH-23390 and haloperidol decreased the locomotor activity in zebrafish larvae. Non-selective dopamine agonists and antagonists showed biphasic effects. Apomorphine—a non-selective dopamine agonist—induced an increase in locomotor activity at lower doses and decreased it at the higher doses. In contrast, butaclamol—a non-selective dopamine antagonist—induced a decrease in locomotor activity at higher doses and increased it at the lower doses [92]. The locomotor behavior induced by these drugs are similar in both mammals and zebrafish larvae [92]. 

Most of the neuroactive drugs that have been studied showed altered locomotor activity in the light-dark locomotion test in zebrafish larvae. In addition, the effects produced by these neuroactive drugs in zebrafish larvae usually confirm the one detected in other higher animals. However, not all neuroactive drugs induced alteration in the locomotor activity or pattern of zebrafish larvae. For instance, sulpiride—a dopamine receptor antagonist—did not alter the movement in zebrafish larvae [99].

### 4.3. Metals, Metallic Ions, and Nanoparticles

The light-dark locomotion test in zebrafish larvae is also used to study the developmental neurotoxicity of metals, metallic ions, and metallic nanoparticles, such as methylmercury, copper, and silver [91,94,95,114]. Methylmercury is a known neurotoxicant and it decreases locomotion in zebrafish larvae [114]. Although the mechanism of action is unknown, this effect is similar to that observed in rodents, where it also decreases the locomotor activity [115] and exploratory activity [116]. Metals such as copper and silver are increasingly used in nanoparticles. The light-dark locomotion test has been utilized to study the neurobehavioral effects of these metals and their nanoparticles in zebrafish larvae. In the light-dark locomotion test, both copper and silver altered the locomotor activity in zebrafish larvae at sublethal concentrations. Copper decreased the locomotor activity in the dark condition and increased it in the light condition. Copper nanoparticles, however, showed an increase in locomotor activity only in the light condition [94,95]. Silver showed a biphasic effect, i.e., it increased the locomotor activity at lower concentrations and decreased it at higher concentrations. Silver nanoparticles decreased the locomotor activity irrespective of the concentrations [91]. Thus, the light-dark locomotion test in zebrafish larvae could be utilized in screening the neuroactivity of metals, their ions, and nanoparticles. 

### 4.4. Environmental Toxicants

Zebrafish larvae are also increasingly used for studying the behavioral toxicity of environmental toxicants [88,89,90,98,102]. The study of toxicants-induced behavior alteration in zebrafish larvae is important as it not only provides their effect in aquatic life, but also gives a clue about the neurobehavioral effects they cause in the broader ecosystem, including humans. The behavior of zebrafish larvae is modulated by the environmental toxicants. The light-dark locomotion test has been applied for studying the effects of several environmental toxicants such as tetrabromodiphenylether (6-OH-BDE-47), chlorpyrifos, perfluorooctane sulphate (PFOS), and dechlorane plus (DP) [89,93,101,102]. Chlorpyrifos and (6-OH-BDE-47) are known neurotoxicants [93] whereas PFOS accumulates in the brain and has potential neurotoxic effects [101]. These neurotoxicants showed altered locomotor activity in zebrafish larvae. Chlorpyrifos and 6-OH-BDE-47 decreased the locomotor activity [89,93,102] and PFOS showed a biphasic pattern with an abrupt increase in locomotor activity followed by a transient decrease before reaching the plateau effect following transition from light to dark [101]. DP decreased the locomotor activity in zebrafish larvae by inhibiting the axonal growth of primary motor neurons [102]. Some environmental toxicants showed neurobehavioral alterations similar to those observed in rodent models. For instance, arsenic showed increased locomotor activity [98] and tributyltin significantly reduced the locomotor activity [103] in zebrafish larvae. These effects were similar to that described in the rodent models where arsenic is shown to cause an increase in locomotor activity [98] and tributyltin is shown to cause a dose-dependent decrease in spontaneous motor activity [117]. Thus, the light-dark locomotion test in zebrafish larvae has been successfully employed for the study of neurobehavioral alterations caused by various environmental toxicants. 

## 5. Conclusions

Zebrafish larvae provide the advantage of performing large-scale high-throughput screening of thousands of neuroactive compounds in a day. The small size, rapid development, easy manipulation, cost-effectiveness, and availability of large numbers of eggs are some of the major advantages of the larvae model compared to the adult zebrafish and other vertebrate models. Zebrafish larvae are gaining popularity for behavioral studies due to their robust behavioral repertoire, similarity to higher vertebrates, and the possibility of conducting high-throughput research. The analysis of locomotor behavior by the light-dark locomotion test is increasingly utilized for the screening of neuroactive compounds. Numerous experiments conducted across different labs using various protocols over the years have shown that the light-dark locomotion test can be successfully used for the screening and analysis of neuroactive compounds. In addition, it could be a complementary test in evaluating and determining the neuroactivity of putative neuroactive compounds. 

## Figures and Tables

**Table 1:** Light-dark locomotion test protocols used by various studies for testing neuroactive compounds.

Compounds	Stage and Well Plate	Protocol	References
Aconitine Pentylenetrazol 4-aminopyridine	4–6 dpf, 48 and 96 well plates	4 successive cycles of 10 min alternating light and dark.	[90]
Ag+ and AgNPs (silver nanoparticles)	5 dpf, 48 well plate	18 alternating dark and light cycles of 5 min each.	[91]
Apomorphine SKF-38393 Quinpirole Butaclamol SCH-23390 Haloperidol	6 dpf, 96 well plate	10 min acclimatization in dark followed by 2 cycles of 10 min of light and 20 mins of dark.	[92]
Bisphenol A (BPA) Tetrabromobisphenol A (TBBPA)	4–5 dpf, 96 well plate	20 mins of light followed by 10 min of dark and 10 min of light.	[88]
Chlorpyrifos 6-hydroxy-2,2’,4,4’ tetrabromodiphenyl ether (6-OH-BDE-47)	6 dpf, 96 well plate	10 min acclimatization followed by 10 min alternating light and dark for 2 times.	[93]
Cocaine Ethanol D-Amphetamine	6 dpf, 96 well plate	20 mins acclimatization in dark followed by 10 min of alternating light and dark for 70 min.	[83]
Copper	5 dpf, 96 well plate	4 successive cycles of 10 min alternating light and dark.	[94]
Copper ions, copper oxide nanoparticles	4 dpf, 24 well plate	18 alternating cycles of 5 min of light and 5 min of dark.	[95]
Diphenylhydantoin	5 dpf, 24 well plate	10 min acclimatization followed by 30 min of light and 5 min of dark.	[52]
Ethanol	6 dpf, 96 well plate	20 mins acclimatization in dark followed by 10 min in dark and 10 min in light, then 20 mins in dark, and then another cycle of 10 min of light and 20 mins of dark.	[84]
Ethanol	6 dpf, 96 well plate	15 min in dark followed by 15 min in light and 15 min in dark.	[96]
Ethanol	9-10 dpf, 24 well plate	5 min acclimatization in light followed by 15 min of dark and 5 min of light.	[97]
Inorganic arsenic	7 dpf, 24 well plate	Acclimatization for 10 min followed by 2 successive cycles of 10 min of light and 10 min of dark.	[98]
MK-801; Pentylenetetrazole; Valproic acid sodium salt; Yohimbine hydrochloride; 5,5-Diphenylhydantoin sodium salt Sulpiride	7 dpf, 24 well plate	60 min in light followed by 5 min in dark. The activities of zebrafish larvae during the last 10 min of the light period and the 5 min of the dark period were analyzed.	[99]
Pentylenetetrazole	5 and 7 dpf, 24 well plate	10 min acclimatization in light followed by 40 min of light and then 3 successive cycles of 10 min of light and 5 min of dark.	[100]
Pentylenetetrazole Picrotoxin	5 dpf, 24 well plate	25 min of acclimatization in environment followed by 5 min in light and 5 min in dark.	[54]
Perfluorooctane sulphate (PFOS)	6 dpf, 48 well plate	15 min acclimatization followed by 10 min in dark and 10 min in light.	[101]
Polybrominated diphenyl ethers and their hydroxyl metabolites (OH-BDEs MeO-BDEs)	5, 6 and 7 dpf, 48 well plate	10 min light adaptation followed by two repeated cycles of 10 min of dark and 10 min of light.	[89]
2,2’,4,4’-Tetrabromodiphenyl ether (BDE-47)	5 dpf, 24 well plate	70 min of alternating 10 min of light and 10 min of dark starting with a light cycle.	[102]
Tributyltin	4 dpf, 96 well plate	50 min of alternating 10 min of light and 10 min of dark starting with a dark cycle.	[103]
Venlafaxine	5 dpf, 96 well plate	Acclimatization for 1 h followed by 60 min of alternating cycles of 7.5 min of light and 7.5 min of dark.	[104]
Yohimbine	5 and 7 dpf, 24 well plate	10 min of acclimatization with light followed by 40 min of light and three 15 min cycles of 10 min of light and 5 min of dark.	[82]
